# FcpB Is a Surface Filament Protein of the Endoflagellum Required for the Motility of the Spirochete *Leptospira*

**DOI:** 10.3389/fcimb.2018.00130

**Published:** 2018-05-08

**Authors:** Elsio A. Wunder, Leyla Slamti, David N. Suwondo, Kimberley H. Gibson, Zhiguo Shang, Charles V. Sindelar, Felipe Trajtenberg, Alejandro Buschiazzo, Albert I. Ko, Mathieu Picardeau

**Affiliations:** ^1^Department of Epidemiology of Microbial Diseases, Yale School of Public Health, New Haven, CT, United States; ^2^Oswaldo Cruz Foundation, Brazilian Ministry of Health, Salvador, Brazil; ^3^Institut Pasteur, Biology of Spirochetes Unit, Paris, France; ^4^Departments of Molecular Biophysics and Biochemistry, Yale University School of Medicine, New Haven, CT, United States; ^5^Institut Pasteur de Montevideo, Laboratory of Molecular and Structural Microbiology, Montevideo, Uruguay; ^6^Department of Microbiology, Institut Pasteur, Paris, France

**Keywords:** *Leptospira*, spirochetes, flagella, motility, flagellar system

## Abstract

The spirochete endoflagellum is a unique motility apparatus among bacteria. Despite its critical importance for pathogenesis, the full composition of the flagellum remains to be determined. We have recently reported that FcpA is a novel flagellar protein and a major component of the sheath of the filament of the spirochete *Leptospira*. By screening a library of random transposon mutants in the spirochete *Leptospira biflexa*, we found a motility-deficient mutant harboring a disruption in a hypothetical gene of unknown function. Here, we show that this gene encodes a surface component of the endoflagellar filament and is required for typical hook- and spiral-shaped ends of the cell body, coiled structure of the endoflagella, and high velocity phenotype. We therefore named the gene *fcpB* for flagellar-coiling protein B. *fcpB* is conserved in all members of the *Leptospira* genus, but not present in other organisms including other spirochetes. Complementation of the *fcpB*^−^ mutant restored the wild-type morphology and motility phenotypes. Immunoblotting with anti-FcpA and anti-FcpB antisera and cryo-electron microscopy of the filament indicated that FcpB assembled onto the surface of the sheath of the filament and mostly located on the outer (convex) side of the coiled filament. We provide evidence that FcpB, together with FcpA, are *Leptospira*-specific novel components of the sheath of the filament, key determinants of the coiled and asymmetric structure of the endoflagella and are essential for high velocity. Defining the components of the endoflagella and their functions in these atypical bacteria should greatly enhance our understanding of the mechanisms by which these bacteria produce motility.

## Introduction

Spirochetes including the agents of leptospirosis (*Leptospira interrogans*), syphilis (*Treponema pallidum*), and Lyme disease (*Borrelia burgdorferi*), have evolved characteristically rapid and powerful swimming capabilities that enable them to rapidly disseminate through connective tissue, blood, and organs. Pathogenic *Leptospira*, which are the causative agents of more than one million severe cases of leptospirosis with approximately 60,000 deaths per year (Costa et al., 2015; Torgerson et al., 2015), can thus reach all organs of intraperitoneally-infected hamsters, including the brain, within 1 h after inoculation (Wunder et al., 2016a). Spirochetes rely on endoflagella or periplasmic flagella (PF) for motility. The spirochete flagellum is organized with an overall similar architecture as the one observed in the extensively studied exoflagella from *Escherichia coli* and *Salmonella enterica*. Such organization can be described as composed of three main structures: the basal body (which functions as a rotary molecular motor, enabling the flagellum to rotate), a flexible hook (coupling the filament and the basal body) and the filament (Pallen et al., 2005). However, the spirochete basal body, located in the inner membrane, does not penetrate the outer membrane with the striking consequence that the hook and filament are assembled within the confined volume of the periplasmic space, between the peptidoglycan layer and the outer membrane. Spirochete flagella are also more complex in terms of protein components as compared to the extracellular appendages of enterobacteria (Zhao et al., 2014). *Leptospira* genomes contain at least 50 genes known to be directly associated with motility, not including those involved in chemotaxis (Fouts et al., 2016). The flagellar filaments from gram-positive and gram-negative bacteria are typically composed of a single protein species, named flagellin (FliC, FljB, FlpA, and homologous isoforms) (Macnab, 2003). A major exception to that rule are some *Vibrio* and *Helicobacter* species, which have sheathed flagella (Qin et al., 2016; Zhu et al., 2017). Spirochetes PFs display an inner core surrounded by a sheath, and are assembled as multi-protein complexes including multiple flagellin homologs (Li et al., 2010) and several additional key proteins (Picardeau, 2017b).

In *Leptospira* one PF is attached at each pole of the cell, extending axially toward the center without overlapping (Picardeau, 2017b). The *Leptospira* genus comprises pathogenic and non-pathogenic species, which are poorly transformable bacteria (Picardeau, 2017a). Nevertheless, several non-motile mutants were described in the last few years by screening a library of random transposon mutants (Lambert et al., 2012), isolation of spontaneous mutants (Fontana et al., 2016; Wunder et al., 2016b), and targeted mutagenesis (Picardeau et al., 2001; Liao et al., 2009). In pathogenic *Leptospira* strains, non-motile mutants displayed virulence-attenuated phenotypes: mutants can persist in the first few days post-infection at a lower burden when compared to virulent wild-type strains, before being cleared from the blood and organs in acute animal models of infection (Liao et al., 2009; Lambert et al., 2012; Fontana et al., 2016; Wunder et al., 2016b). Such mutants are also not able to translocate across polarized mammalian cell monolayers or the conjunctival membrane in animal models (Wunder et al., 2016b), demonstrating that motility is essential for pathogenesis. Studies of these non-motile mutants also increased our understanding of the molecular architecture of the endoflagellum.

The leptospiral flagellar filament is an assembly of at least 7 proteins: FlaB1-4, FlaA1-2, and FcpA. Deletions of FlaB1, of FlaA1/FlaA2, and of FcpA have a profound effect on cell morphology, motility, flagellar structure, and/or assembly of other flagellar proteins (Picardeau et al., 2001; Lambert et al., 2012; Wunder et al., 2016b). In this study, we identified a novel component of the flagellar filament, FcpB, by screening a library of random mutants in *L. biflexa*. Taken together, our data demonstrate that the flagellar filament is composed of at least 8 proteins and that FcpB is crucial for the ability of *Leptospira* cells to generate high velocity during translational motility.

## Materials and methods

### Strains and growth conditions

*L. biflexa* serovar Patoc strain Patoc 1 (Paris) wild-type, *fcpB*^−^ mutant, and *fcpB*^−/+^ complemented strains were grown in Ellinghausen–McCullough–Johnson–Harris (EMJH) liquid medium or on solid medium containing EMJH supplemented with 1% agar, at 30°C. When necessary, media was supplemented with 50 μg/mL of kanamycin or spectinomycin (Sigma). For growth curves, cell densities were counted in a Petroff-Hausser counting chamber under dark-field microscopy, and triplicate values were recorded for each time point. Replicate averages of cell density were compared between strains at each time point using a two-way repeated measures ANOVA with Bonferroni post-tests (Prism 6, GraphPad Software).

### Random transposon mutagenesis

A *L. biflexa* library of mutants was generated by random transposon insertion mutagenesis as previously described (Slamti and Picardeau, 2012). Briefly, the shuttle vector pCjTKS2 carrying *Himar1* transposon was introduced into *L. biflexa* strain Patoc by conjugation with *E. coli* strain β2163. Kanamycin-resistant colonies were picked and recovered in liquid EMJH medium in 96-well microtiter plates. After 3 days culturing at 30°C, the library was replicated onto solid EMJH medium. The library was subsequently screened for small colonies after 5 days of incubation. DNA was isolated using a Maxwell 16 Cell DNA Purification Kit and Maxwell 16 Instrument (Promega, Madison, WI, USA). To amplify the region surrounding the site of random insertion of the *Himar1* transposon, a two-step, nested, semi- random PCR was performed as previously described (Slamti and Picardeau, 2012). DNA sequencing was performed by Eurofins MWG Operon (Atlanta, GA), and sequences were aligned using MaGe (http://www.genoscope.cns.fr/agc/microscope/home/index.php).

### Genetic complementation

The *fcpB* gene together with its native promoter was amplified from genomic DNA of *L. biflexa* serovar Patoc strain Patoc 1 (LEPBIa1597) and *L. interrogans* serovar Copenhageni strain Fiocruz L1-130 (LIC11848) and cloned into pCjSpLe94 (Picardeau, 2008). Plasmid constructs were introduced in the *fcpB*^−^ mutant by conjugation with *E. coli* β2163 carrying the plasmids as previously described (Picardeau, 2008). The transformed bacteria were plated onto EMJH plates containing 50 μg/mL of spectinomycin and incubated at 30°C for 1 week. Colonies were then inoculated into liquid EMJH supplemented in spectinomycin for further analysis.

### Purification of periplasmic flagella

Purified PFs were obtained as previously described (Fontana et al., 2016; Wunder et al., 2016b). Briefly, late-logarithmic-phase cells were cultured in 300 mL of liquid EMJH media and harvested at 8,000 g at 4°C for 20 min, washed with 1X PBS without divalent cations (Sigma), centrifuged at 8,000 g at 4°C for 15 min, washed with sucrose buffer (0.5 M sucrose, 0.15 M Tris, pH 8.0), centrifuged as before, and resuspended in 15 mL sucrose buffer. Cells were stirred on ice for 10 min, treated with Triton X-100 (Sigma) to a final concentration of 1%, stirred at room temperature for 30 min, then treated with lysozyme (10 mg/mL) drop-wise with stirring at room temperature. EDTA (pH 8.0) was added drop-wise reaching 2 mM, always stirring at room temperature for 2 h. 300 μL of a 0.1 M MgSO4 solution was added with stirring for 5 min, followed by 300 μL of 0.1 M EDTA (pH 8.0) with stirring for 5 min. Cells were centrifuged at 17,000 g at 4°C for 15 min, and the supernatant was transferred to a clean 50 mL polypropylene tube. 2 mL of 20% PEG-8000 in 1M NaCl was added, and tubes were mixed thoroughly and kept on ice for 30 min. Samples were centrifuged at 27,000 *g* at 4°C for 45 min, the supernatant was discarded, and the pellet resuspended in 3 mL ddH_2_O in 1X PBS. Periplasmic flagella were pelleted by ultracentrifugation at 80,000 g at 4°C for 45 min. The supernatant was discarded and pellets containing purified periplasmic flagella were resuspended in ddH_2_O. Protein concentrations were measured using a Bradford assay (Bio-Rad) on a Thermo Scientific 60S UV-Vis Spectrophotometer and samples were stored at 4°C.

### Electron microscopy

Carbon film copper 300 mesh grids (Electron Microscopy Sciences) were glow-discharged at 25 mA for 25 s at 10^−1^ mbar in a Bal-Tec SCD 005 Sputter Coater. Glow-discharged grids were applied to a 15 μL drop of purified periplasmic flagella for 5 min, washed in 3 drops of ultrapure water, and negative-stained with 2% phosphotungstic acid (PTA, adjusted to pH 7) (Electron Microscopy Sciences) for 2 min. Grids were wicked from behind with Whatman filter paper and allowed to dry horizontally for 20 min before imaging in a Philips TECNAI 12 BioTwin II electron microscope at 80 kV. Images were acquired on a Soft Imaging System Morada camera using iTEM image acquisition software. Diameter measurement of PFs was performed using Adobe Illustrator software.

### CryoEM data collection and image analysis

Purified wild-type or *fcpB*^−^ flagella were applied to glow-discharged Quantifoil holey carbon grids, blotted and plunge-frozen using a Vitrobot Mark III (FEI Company, Eindhoven, The Netherlands). Cryo-EM images were collected using a Tecnai F20 transmission electron microscope (FEI Company) operating at 200 kV. Micrographs were acquired at ~52 KX magnification in SerialEM (Mastronarde, 2005) using an UltraScan 4000 (4 K by 4 K) CCD camera (Gatan, Inc., Pleasanton, CA, USA) at the Yale School of Medicine, Center for Cellular and Molecular Imaging. Box segments of imaged filaments were manually selected using the *boxer* program from the *EMAN* software suite (Tang et al., 2007). CTF-correction was carried out on the selected particles using Gctf (Zhang, 2016) and then subjected to iterative reference-free 2D classification and alignment using RELION (Scheres, 2012), with *N* = 50 classes. Following this step, a subset of class averages with well-resolved features were identified for both wild-type (29) and *fcpB*^−^ mutant (13) samples, and in each case the majority of class averages (~19/29 WT; ~8/13 *fcpB*^−^ mutant) were nearly indistinguishable from the examples shown in **Figure 6E**. The highly limited number of distinct views of the flagellum derived from 2D classification is likely due to the constrained orientation of the curved filament within the ice layer.

### Motility assays

Bacteria were counted in a Petroff-Hausser counting chamber under dark-field microscopy. A total of 10^5^ leptospires in 5 μL were inoculated onto soft 0.5% agar EMJH plates and plates were incubated at 30°C for 5 days. Plates were imaged in a Bio-Rad ChemiDoc XRS+ photo documentation instrument with white light transillumination using ImageLab 3.0 software (Bio-Rad), and the diameter of the zone of spread for each colony was measured at the pixel level in Photoshop CS 5.5 Standard (Adobe). Statistical analysis was performed using a one-way ANOVA with Bonferroni multiple-comparisons post-test (Prism 6.3, GraphPad Software).

### Immunoblotting and immunogold labeling

FcpB from *L. interrogans* (LIC11848) was expressed and purified in *E. coli*, and rabbit polyclonal antibodies were raised against the purified recombinant FcpB (Cocalico Biological, Inc). Western blots were performed by transferring the proteins (flagella preparations) from the gels to nitrocellulose membranes. Rabbit anti-FcpA (1:1000), anti-FcpB (1:2000), and anti-FlaA2 (1:1,000) antibodies were added. Secondary detection was performed with an HRP-conjugated goat anti-rabbit secondary antibody (1:100,000), and images were acquired in a Bio-Rad ChemiDoc XRS+ photo documentation instrument.

The immunogold labeling was performed as previously described (Wunder et al., 2016b). Purified PFs were allowed to absorb for 10 min on glow-discharged, formvar-coated, 300-mesh copper grids. Grids were blocked with 0.1% BSA for 2 min and incubated with anti-FcpB primary antibody (1:10 dilution) for 20 min. Grids were washed three times with ultrapure water and blocked with 0.1% BSA for 2 min. Anti-rabbit 5 nm gold-conjugated Protein A (PAG) was used at 1:50 dilution and incubated for 20 min. After 3 washings as described above, grids were negatively stained with 2% PTA pH 7. Grids were observed using a transmission electron microscope as described above.

### Dark-field video microscopy

Late-log phase cultures were diluted to 1:100 in 0.1% sodium azide (Sigma) or 1:10 in 1% methyl-cellulose (MP Biomedicals) and 10 μL of the dilution was transferred to a slide. An 18 × 18 mm coverslip was applied, and the edges of the coverslip were sealed with nail polish. Samples were analyzed in an Axio Imager.M2 motorized dark-field microscope (Carl Zeiss). Videos were recorded on an AxioImagerM3 camera (Carl Zeiss) at 1,388 × 1,040 standard mono pixels in 16-bit gray levels using AxioVision 4.8.2 software (Carl Zeiss). Samples were focused 5–10 μm beneath the glass coverslip (z-position), and the field of view for each new video (xy-position) was systematically selected so as to be non-redundant. Videos for qualitative analysis were recorded at 100X (EC Plan-Neofluar 100x/1.30 Oil) under oil immersion (Zeiss Immersol 518F) in 33 ms exposures at 125 ms intervals over 10 s (frame rate 8 fps; digital gain = 2). Digital high-speed videos for tracking were recorded at 200 ms intervals for up to 10 s (50 frames at 5 fps; digital gain = 1; sensitivity = 100%; image orientation = flipped vertically). All videos recorded were analyzed using the AxioVision Tracking Module. Seeds (search area = 30) were manually set on the start frame of each video, noise reduction was applied (noise reduction = 5), and automatic tracking was performed of each seed by the software algorithm.

Inclusion criteria for tracking consisted of all leptospires whose search area was entirely within the field of view at the start frame and in the plane of focus at the start frame. Chains of two or more leptospires (for example, dividing cells) were excluded from tracking, as were leptospires whose tracks jumped to nearby static objects (for example, debris or imperfections on the surface of the glass slide) and could not be followed by the tracking algorithm. The instantaneous velocity of each tracked leptospire was recorded by the tracking software at each frame by comparison to the preceding frame, and the mean velocity for each leptospire was calculated by averaging the instantaneous velocities of that particular leptospire and reported by the software as a mean velocity for each of the individual leptospires tracked in a given video. Because the method that the software employs to calculate the mean velocity for each cell produces a final value that varies with the cumulative distance traveled by the cell rather than the total vector displacement of the cell, we are aware that this calculation more accurately reflects average “speed” as opposed to “velocity.” However, for consistency with the language used by the tracking software, here we will continue to refer to this calculation as “mean velocity.”

### Statistical analysis and software

Statistical analyses were performed in Prism 6.3 (GraphPad Software). Drawings were composed in Illustrator CS 5.5 (Adobe), graphs were generated in Prism 6.3, and tables and figures were assembled in InDesign CS 5.5 (Adobe). Gel and blot images were captured and exported using ImageLab 3.0 (Bio- Rad). Dark-field micrographs and videos were captured using AxioVision 4.8.2 (Carl Zeiss) and tracking analysis was performed using the Tracking module available for AxioVision from Zeiss.

## Results

### Identification of an *fcpB* non-motile mutant

**Figure 1:**
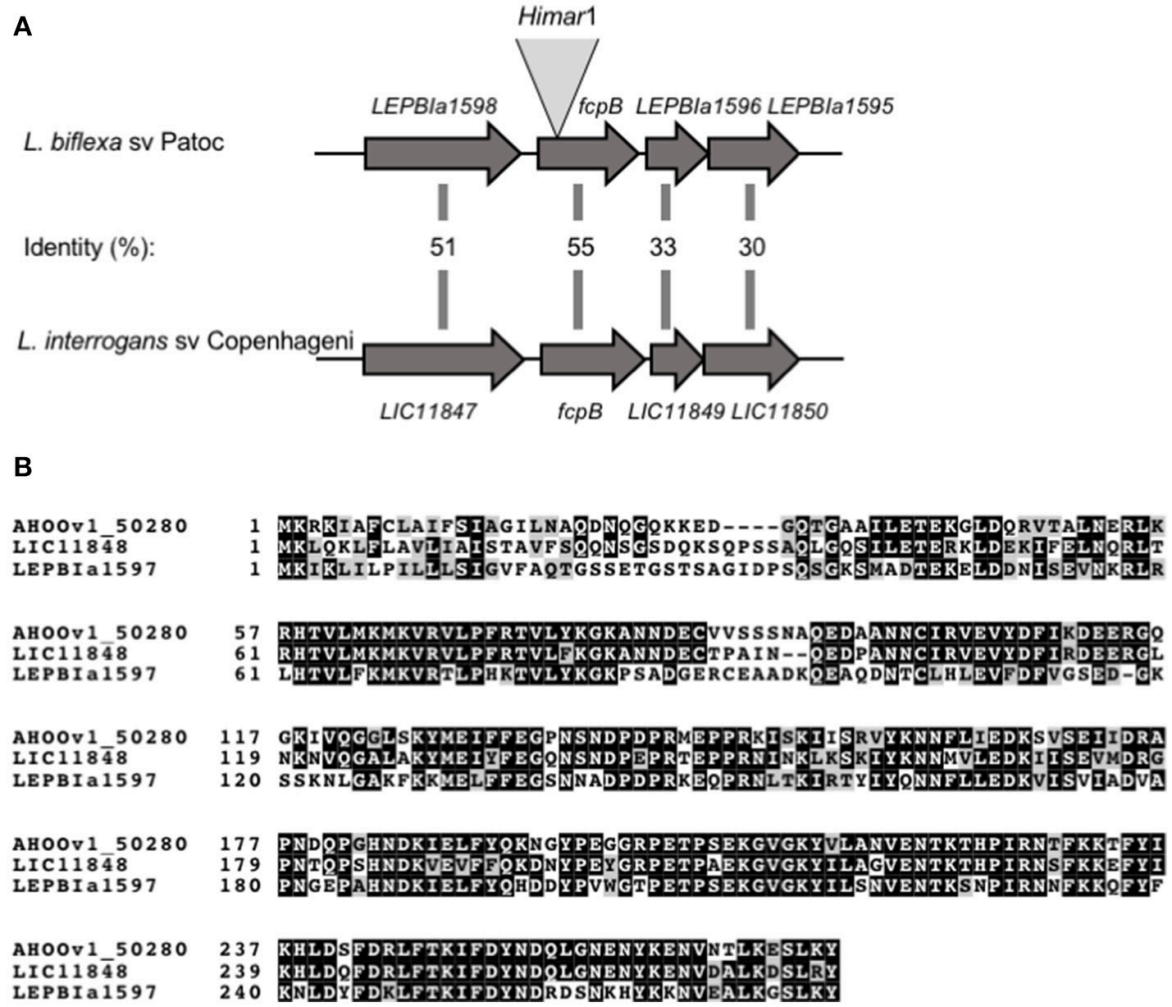
Schematic representation of *fcpB* in *Leptospira* spp. **(A)** Genetic locus of *fcpB* in *L. biflexa* (LEPBIa1597) and *L. interrogans* (LIC11848) with the corresponding amino acid identities between the two species. The insertion site of *Himar1* in the chromosome of the *fcpB*^−^ mutant is indicated. **(B)** Alignment of FcpB protein sequences from the saprophyte species *L. biflexa* (LEPBIa1597), the intermediate *L. licerasiae* (AH00v1_50280), and the pathogenic *L. interrogans* (LIC11848). Identical and similar residues are shaded in black and gray, respectively.

**Figure 2:**
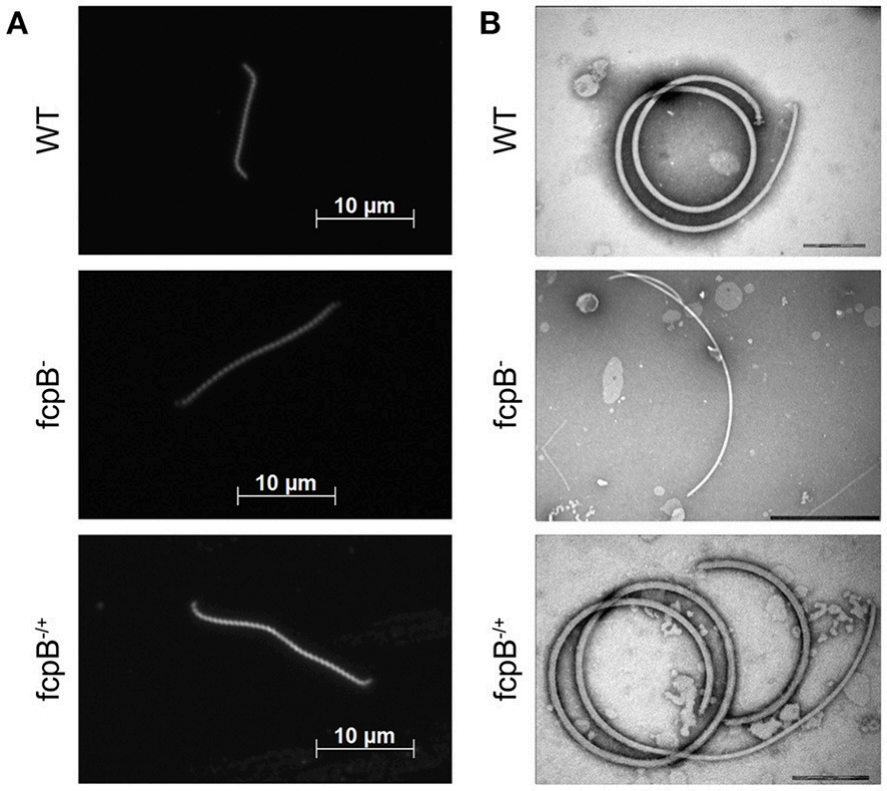
Determination of phenotypes in cell morphology and purified flagella caused by deletion of *fcpB*. **(A)** The *L. biflexa fcpB*^−^ mutant is deficient in its ability to form hook- and spiral-shaped ends, which are characteristic of wild-type cells. Cells were examined by dark-field microscopy with a 100X oil-immersion objective. **(B)** Periplasmic filaments purified from the *L. biflexa fcpB*^−^ mutant lack the super-coiled morphology, which is characteristic of purified wild-type flagellar filaments when examined *in vitro*. Complementation of *fcpB*^−^ mutant restores wild-type morphology and coiled flagellar filament structure. Bar scales for negative staining images are 200 nm for WT and *fcpB*^−/+^ complemented strain, and 500 nm for *fcpB*^−^ mutant.

A library of 3,500 random mutants was screened for possible motility-deficient phenotypes by identifying clones forming small colonies on agar plates. In total, 172 mutants forming small colonies were picked for subsequent identification of the *Himar1* transposon insertion site. Dark-field microscopy analysis showed that one particular mutant lacked the distinctive hook-shaped ends of the cell (Video 2), compared to wild-type (Video 1). This mutant had transposon insertion at nucleotide position 37 of the *LEPBIa1597* gene (Figure 1A), which encodes a conserved hypothetical protein without predicted function and not genetically linked to any other flagellar gene. *LEPBIa1597* encodes a putative N-terminal signal peptide and is conserved among all *Leptospira* species, including in intermediates and pathogens; although the sequence homology is lower for the first 35 predicted amino acids (Figure 1B). Complementation of such *LEPBIa1597*^−^ mutant with the respective wild-type gene from *L. biflexa* or with the orthologous gene from *L. interrogans* (data not shown), restored the morphology (Figure 2A) and motility (Video 3) comparable with the wild-type Patoc strain.

### FcpB is required for hooked-end morphology, coiled PF, and normal motility

The *LEPBIa1597*^−^ mutant presented an atypical straight cell morphology under dark field microscope, lacking the characteristic hook-shaped extremities; even though bent ends could be observed, they were not as pronounced as the spiral-shaped ends of wild-type strains (Figure 2A). To further investigate the function of the protein encoded by *LEPBIa1597*, flagella were purified and observed by electron microscopy. Wild-type flagella typically form a strongly supercoiled filament structure characteristic of *Leptospira* Li et al. (2010). Notably, purified PFs from *LEPBIa1597*^−^ do not form supercoils (Figure 2B), suggesting a similar phenotype as the *fcpA*^−^ mutants that we have previously characterized Wunder et al. (2016b). We have thereby decided to name *LEPBIa1597* as *fcpB* (for flagellar-coiling protein B). We did not observe significant differences (*p* = 0.09) in flagellum diameters between wild-type and *fcpB*^−^ mutant strains (20.6 ± 0.64 nm and 19.9 ± 1.06 nm, respectively).

**Figure 3:**
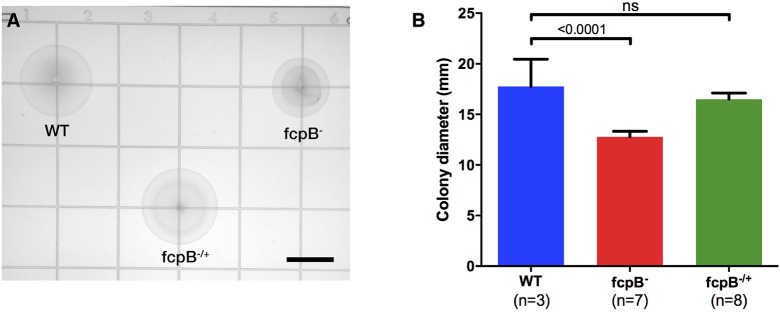
Motility phenotype of the *fcpB*^−^ mutant in semisolid media. Soft agar plates were inoculated with 10^5^ leptospires of the wild-type, *fcpB*^−^ mutant, and *fcpB*^−/+^ complemented strains. **(A)** The diameter of the zone of spread for each colony was measured using image analysis software. Bar scale, 10 mm. **(B)** The average colony diameter measured for the *fcpB*^−^ mutant was significantly smaller (*p* < 0.0001) than for the wild-type. The difference in average colony diameter between the *fcpB*^−/+^ complemented and wild-type strains was not significant.

Analysis of bacterial motility on soft agar plates showed a significant reduction of motility of the *fcpB*^−^ mutant compared to the wild-type and *fcpB*^−/+^ complemented strains in this medium (Figure 3A). The average colony diameter measured for wild-type was 17.8 ± 2.7 mm (average ± SD, *N* = 3). The average colony diameter measured for the *fcpB*^−^ mutant was smaller (12.8 ± 0.6 mm, *N* = 7) and was significantly different from wild-type (*P* < 0.0001, one-way ANOVA with Bonferroni multiple-comparisons post-test; Figure 3B). Complementation of *fcpB*^−^ mutant restored wild-type colony size (16.5 ± 0.6 mm, *N* = 8), and no statistically significant difference was observed between the average colony diameter of the *fcpB*^−/+^ complemented strain and the wild-type (α = 0.05, one-way ANOVA with Bonferroni multiple comparisons post-test; Figure 3B). Because the inoculum was standardized and FcpB does not affect bacterial growth kinetics (data not shown), the difference in average colony diameter between the wild-type and mutant can be attributed to a difference in motility in semisolid media rather than a difference in growth kinetics.

**Figure 4:**
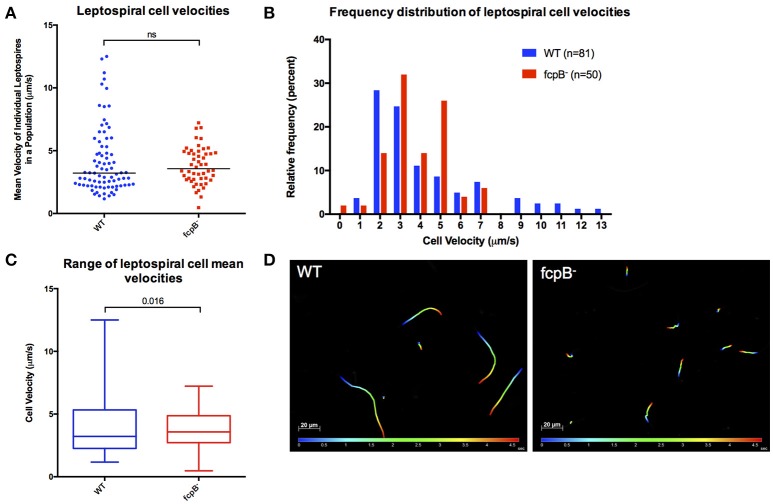
Analysis of velocity of the *fcpB*^−^ mutant cells in liquid viscous media. Dark-field video microscopy and tracking analysis were used to measure the average velocities of individual cells in viscous liquid media containing 1% methyl-cellulose. **(A)** Scatter plot of the velocities of individual cells shows that the difference in median velocity (horizontal bar) between the wild-type and mutant populations is not statistically significant. **(B)** Histogram showing the relative frequency of the velocities of individual cells. Note that depletion of FcpB affects the proportion of highly motile leptospires in a population. **(C)** The range of leptospiral cell velocities in a population is reduced in the *fcpB*^−^ mutant, with a lower maximum velocity, when compared to the wild-type strain. **(D)** Representative trajectories of wild-type and *fcpB*^−^ mutant over 5 s, illustrating that the *fcpB*^−^ mutant population is deficient in highly-motile leptospires.

To determine whether FcpB affects motility in viscous liquid media, we performed dark field video microscopy of cells in late log phase in media containing 1% methyl-cellulose. Analysis of the average velocities recorded for each individual leptospire in a population (Figure 4A) showed that the median cell velocities in the wild-type (3.2 μm/s, *N* = 81) and *fcpB*^−^ mutant (3.6 μm/s, *N* = 50) populations were not significantly different (Kruskal-Wallis test, α = 0.05). However, the *fcpB*^−^ mutant population is specifically deficient in highly-motile leptospires (>7 μm/s) (Figure 4B and Videos 1–3). Further analyses showed that although the 25th and 75th percentile of average cell velocities within the wild-type and mutant populations are similar, the wild-type population has a significantly higher maximum range of cell velocities in viscous liquid media compared to *fcpB*^−^ mutant cells (15 and 2%, respectively, *p* = 0.016; Figures 4A–C). Representative color tracks (Figure 4D) recorded over 5 s in viscous liquid media show that a wild-type population appears to have a greater range of translational cell velocities compared to the *fcpB*^−^ mutant.

### FcpB is associated with the surface of the endoflagellum filament

**Figure 5:**
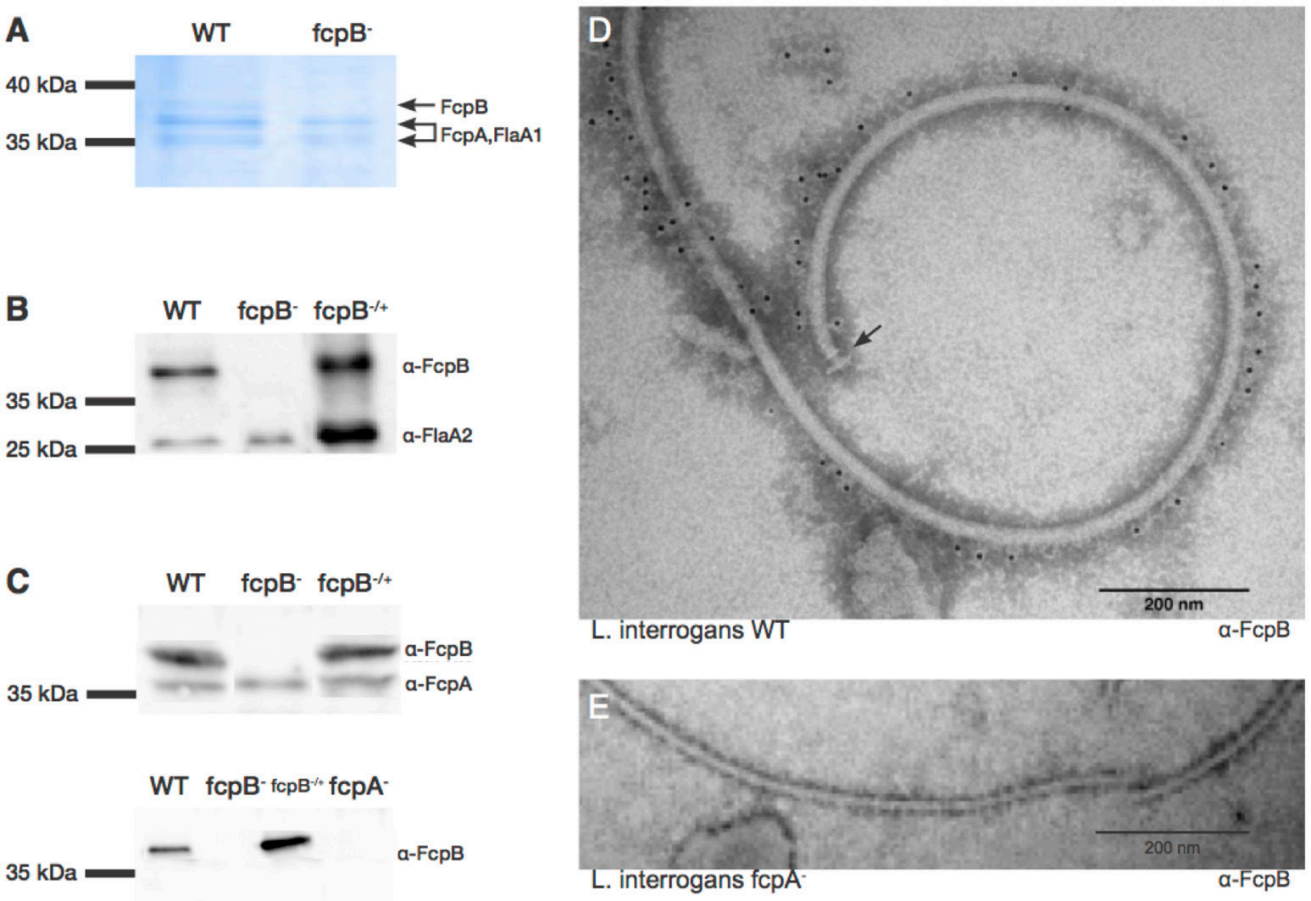
Characterization and localization of FcpB in *Leptospira* cells. **(A)** Coomassie Blue-stained SDS-PAGE gel of purified periplasmic flagella. FcpA, FcpB, and FlaA1 are indicated, according to MS-MS identification. **(B)** Western blot of purified flagella using anti-FcpB antibodies showing that FcpB is expressed in wild-type and *fcpB*^−/+^ complemented strains, but not in the *fcpB*^−^ mutant. Anti-FlaA2 antibodies were used as a positive control. **(C)** Western blots of whole-cell lysates using anti-FcpA and anti-FcpB antibodies, showing that FcpB is not detected in the *L. biflexa fcpA*^−^ mutant. **(D)** Immunogold labeling with anti-FcpB antibody on negatively-stained periplasmic flagella purified from wild-type *L. interrogans*, showing that FcpB is surface-exposed on the supercoiled flagellar filament and is distributed all along its length. The flagellar basal body is labeled with an arrow. **(E)** Immunogold labeling with anti-FcpB antibody on negatively-stained periplasmic flagella purified from a *L. interrogans fcpA*^−^ mutant, detecting no FcpB.

Based on the results described above, we hypothesized that FcpB may be a novel structural component of the PF in *Leptospira* spp. To determine whether FcpB was associated with the flagellar filament, purified PFs were analyzed by SDS-PAGE. We observed that FcpB from *L. biflexa* consistently runs as a higher apparent molecular weight species (~37 kDa) compared to its predicted size (31.4 kDa) on denaturing polyacrylamide gels. Such ~37 kDa band is noticeably absent in purified PFs from the *fcpB*^−^ mutant (Figure 5A). Western blotting of purified PFs and reaction with anti-FcpB antibodies confirmed that the ~37 kDa band in wild-type purified PFs indeed corresponds to FcpB (Figure 5B), demonstrating that FcpB is absent in the *fcpB*^−^ mutant strain but present in flagella purified from the *fcpB*^−/+^ complemented strain (Figure 5B). The flagellar filament protein FlaA2 was used as a positive control throughout. Interestingly, FcpB from *L. interrogans* migrated on SDS-PAGE as expected from its predicted molecular weight of 32 kDa (data not shown), suggesting that *L. biflexa* FcpB, but not *L. interrogans* FcpB, may be post-translationally modified by methylation, acetylation, phosphorylation or glycosylation (Stewart et al., 2015).

Western blotting of whole-cell lysates from the wild-type, *fcpB*^−^ mutant and *fcpB*^−/+^ complemented strains, showed that in the absence of FcpB, FcpA is still expressed at normal levels (Figure 5C, upper panel). In contrast, when the flagellar sheath protein FcpA is not expressed, FcpB is also absent within the PFs (Figure 5C, lower panel). These results also rule out non-specificity of our anti-FcpB antibody, particularly concerning potential cross-reaction with FcpA.

To determine whether FcpB is surface-exposed on the periplasmic flagellum, we performed immunogold labeling using anti-FcpB antibodies on negative-stained PF purified from wild-type *L. interrogans*. The gold labeling seen on a representative electron micrograph (Figure 5D) demonstrates that FcpB is surface-exposed on the periplasmic flagellum and is distributed all along the length of the flagellum. The same immunogold labeling experiment performed on PF purified from an *L. interrogans fcpA*^−^ mutant shows no labeling of FcpB on the periplasmic flagellum (Figure 5E), consistent with the Western blot results (Figure 5C), once again confirming that FcpB is not expressed on flagella that lack the flagellar sheath protein FcpA. Surprisingly, the vast majority of FcpB labeling was found in the outside of the curved filament (Figure 5D).

**Figure 6:**
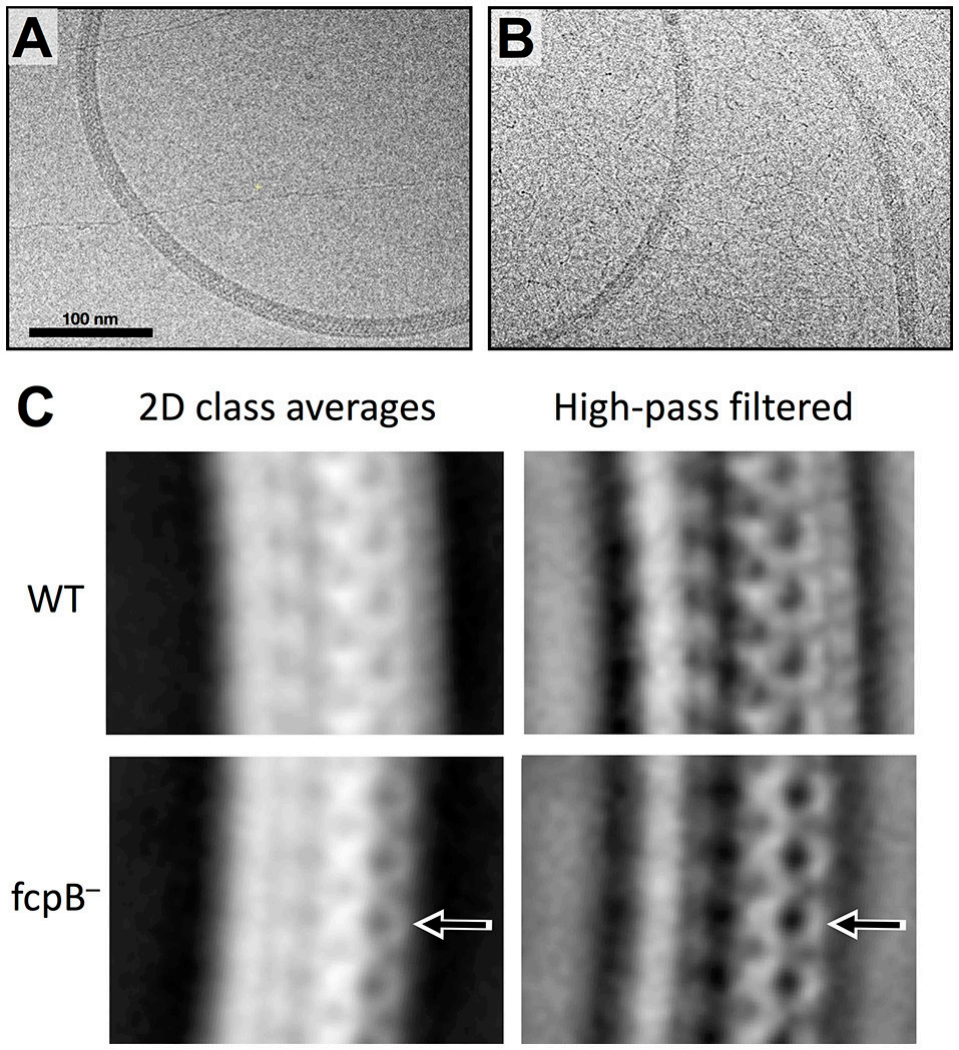
Cryo-EM imaging reveals morphologic differences near the outer diameter of curved flagellar filaments. **(A)** Example micrograph of purified wild-type *Leptospira* flagella, exhibiting a characteristic coiled shape. **(B)** Example micrograph of flagella purified from the *fcpB*^−^ mutant. **(C)** 2D class averages representing the dominant views of flagella found in wild-type (top) and *fcpB*^−^ mutant (bottom) of *L. biflexa* cells. Periodic features corresponding to a subunit repeat distance of ~52 Å are apparent. Arrows denote density features present near the outer diameter of the wild-type flagella that are not seen in the *fcpB*^−^ mutant flagellum. To better visualize fine structural details, the right-hand panels depict high-pass filtered (100 Å) versions of the same class averages.

Although the diameter measurement didn't show any difference, a detailed analysis of the *L. biflexa* wild-type (Figure 6A) and *fcpB*^−^ mutant (Figure 6B) filaments by cryo-electron microscopy revealed additional insights on the morphology of the filament: the diameter of the *fcpB*^−^ mutant filament is slightly reduced in comparison to the wild-type filament (~170 Å vs. ~200 Å), due to density missing from the outer (convex) side of the *fcpB*^−^ mutant filament (Figure 6C), consistent with the FcpB immunolabeling experiments reported above (Figure 5).

## Discussion

We demonstrated that FcpB is a novel component of the PF sheath for the *Leptospira* spirochete, and that this protein, together with FcpA, determines the cell shape, and in turn, the ability of the spirochete to generate full translational motility. *Leptospira*, which includes both pathogenic and free-living species, represent the simplest model to study the spirochetal motility machinery, with only one flagellum attached at each pole of the cell. Previous studies suggested that the PF of the spirochete is composed of a core and a sheath formed by FlaB and FlaA proteins, respectively (Charon and Goldstein, 2002). *Leptospira* spp. possess four flagellin-like FlaB isoforms and inactivation of the gene encoding for one of the most abundant FlaB isoforms results in an aflagellated mutant (Picardeau et al., 2001), strongly suggesting that FlaB is essential for full assembly of the filament. *Leptospira* has two distinct FlaA proteins, FlaA1 and FlaA2, and we have previously shown that the *flaA1* and *flaA2* mutants were able to assemble PFs of wild-type diameter, but the coiled structure of the filament was affected, depending on whether FlaA1 or both FlaA proteins were expressed (Lambert et al., 2012). These data indicate that FlaA's are not required for sheath formation, and that the sheath is likely composed of other proteins instead or in addition to the FlaAs.

Our most recent studies using whole genome sequencing of a spontaneous non-motile strain allowed for the identification of a novel flagellar protein that we named FcpA (Wunder et al., 2016b). This protein is present in high copy number in *L. interrogans* (8,021 copies per cell) (Malmström et al., 2009) and diameters of the filaments of FcpA-depleted cells are significantly smaller (15 nm) when compared to the wild-type strains (21 nm) (Wunder et al., 2016b). Taken together, these data are consistent with FlaB1-4 forming the filament core, while FcpA, possibly in tandem with FlaA1 and FlaA2, is a major component of the sheath. Conservation of the flagellar components in saprophytic and pathogenic *Leptospira* strains (Fouts et al., 2016) makes of the saprophyte *L. biflexa*, an ideal model to understand spirochete motility, highlighting that this species is relatively easy for *in vitro* culturing and genetic manipulations compared to pathogenic species (Picardeau, 2017a). In this study, following the screening of a library of random mutants in *L. biflexa*, we identified and characterized a novel component of the flagellar filament of *Leptospira*. which we have named flagellar-coiling protein B (FcpB).

FcpB is required for the hook-shaped ends of *Leptospira* cells, and also for the strongly super-coiled filaments. The *fcpB*^−^ mutant was morphologically distinct in its inability to form hooked ends. Furthermore, PF purified from the *fcpB*^−^ mutant were unusually straight and lacked the tightly-wound coiling that is typical of wild-type flagella. A key feature of currently accepted biophysical models of *Leptospira* motility, is indeed a highly curved and stiff flagellar filament (Wolgemuth, 2015; Takabe et al., 2017). The coiled flagella in the periplasm are believed to be responsible for bending the ends of the bacterium into either a hook or spiral shape. Thus, the counter-clockwise rotation of the PF is posited to cause the spiral-shaped end morphology, whereas the hook-shaped end occurs when the flagellum is not rotating or is rotating in the clockwise direction. When both ends are hook-shaped or spiral-shaped, the cell appears to flex in place and does not exhibit effective translational motility. When the anterior end is spiral-shaped and the posterior end is hook-shaped the cell moves forward like a rotating screw, presumably due to forces that act between the PF and the cell body. Mutants that lack super-coiled PFs such as the *fcpA*^−^ and *fcpB*^−^ mutants are unable to form hook-shaped ends. However, the motility and morphology phenotypes of the *fcpB*^−^ mutant seem to be less pronounced than the *fcpA*^−^ mutant, as the *fcpB*^−^ mutant is still able to translate in viscous liquid media. This finding indicates that mutants can achieve low velocity motility without having an intact coil filament.

The lack of *fcpB* expression results in a reduction of the range and proportion of highly motile leptospires in a population. The *fcpB*^−^ mutant exhibited significantly decreased motility in semisolid media and qualitatively different translational motility in viscous media, identified by the lack of cells with the ability to produce high velocity. Previous studies have shown that the gyrating spiral-shape on the leading end is sufficient to generate translational motility, since leptospires are still able to move translationally when the trailing hooked-end is prevented from gyrating by attachment to a large bead; the gyration of the hook on the trailing end of the cell does not appear to generate appreciable thrust on its own but is instead thought to provide counter-torque for the leading end of the translating cell (Goldstein and Charon, 1988, 1990). These observations are consistent with our results showing that the *fcpB*^−^ mutant, which is unable to form hooked ends, is still able to translate in viscous liquid media using a form that most resembles a spiral shape on the leading end of the cell. Unfortunately, we could not evaluate the role of FcpB in pathogenic leptospires because our attempts to construct a mutant in pathogenic leptospires were not successful. However, recent studies confirmed the role of motility for the virulence of the agent (Liao et al., 2009; Lambert et al., 2012; Fontana et al., 2016; Wunder et al., 2016b), hence it is reasonable to believe that the lack of FcpB would affect the ability of the bacteria to cause disease.

FcpB is located on the outer surface of the flagellar sheath of *Leptospira*. FcpB was detected in purified PF and was localized with immunogold electron microscopy, showing that FcpB is accessible and distributed along the length of the PF of *L. interrogans*. Furthermore, FcpB contains a putative N-terminal signal peptide, which is consistent with our hypothesis that this protein is exported to the periplasmic space by the *sec* secretion system, where it interacts with the periplasmic flagellar filament. Interestingly, when the flagellar sheath protein FcpA is not expressed, FcpB is not detected, while the opposite is not true. Taken together, these results suggest that FcpB is located on the outer surface of the flagellar sheath protein FcpA, the latter being needed to recruit FcpB onto the filament. Interestingly, FcpA in purified PFs is still accessible to anti-FcpA antibodies, even when FcpB is present (Wunder et al., 2016b), suggesting that FcpB does not form an outer layer completely surrounding FcpA. Although FcpA and FcpB seem to share a similar spatial organization on the filament, FcpA is present in higher copy number than FcpB (8,021 vs. 4,271 copies per cell, respectively) (Malmström et al., 2009) and the *fcpA*^−^ mutant exhibits a thinner filament diameter compared to both wild-type and *fcpB*^−^ mutant strains (15 nm *vs*. 21 nm, respectively), suggesting that FcpA is a major structural component of the filaments' sheath in *Leptospira*. High resolution structural studies of individual filament proteins (San Martin et al., 2017) as well as of the whole supramolecular assembly, are being actively pursued, in order to address these open questions. It will be particularly important to establish the symmetry of the filament architecture and the precise way in which the different protein species get assembled and eventually recruit additional components. One interesting observation from this work is that FcpA and FcpB do not seem to be symmetrically distributed along the filament. The immunogold experiments on purified PFs imply that both FcpA (Wunder et al., 2016b) and FcpB are located mainly on the convex side of the curved super-coiled filament. Cryo-electron microscopy further confirmed a missing density in the convex side of the *fcpB*^−^ mutant filament, indicating an asymmetric localization of FcpB. Such asymmetric organization is atypical for filament proteins like flagellins, which instead self-assemble into helically symmetric structures in exoflagellated bacteria (Yonekura et al., 2003).

In this work, we demonstrated that the novel flagellar protein FcpB is required for wild-type motility, cell morphology, and flagellar architecture in *Leptospira*, where FcpB is associated with the surface of the flagellar filament. Complementation of *fcpB*^−^ mutant qualitatively restored the wild-type morphology of the cell and the PFs, as well as the normal translational motility in viscous liquid media. The complementation quantitatively restored motility in semisolid media to a level that was statistically indistinguishable from that of the wild-type. These findings, together with the recent characterization of FcpA (Wunder et al., 2016b) and FlaA (Lambert et al., 2012) proteins, indicate not only that several filament proteins influence the coiled structure of PFs and allow for normal translational motility, but also confirm that the flagellar structure in *Leptospira* and spirochetes in general, is a much more complex machinery than anticipated. Crystal structures of FcpA (San Martin et al., 2017), FcpB and other flagellar proteins, and 3D electron microscopy approaches to observe the entire flagellum will shed light into the detailed architecture of spirochetal endoflagella. Further examination of their structure by covalent cross-linking and co-immunoprecipitation studies, is expected to uncover additional spatial restraints to produce an accurate 3D model of the supramolecular assembly. Solving the puzzle of how these atypical bacteria move shall contribute to discerning fundamental aspects of spirochete biology that underlie their ability to cause disease.

## Author contributions

EW, LS, CS, FT, AB, AK and MP conceived and designed the experiments. EW, LS, DS, KG and ZS performed the experiments. CS, AB, AK and MP contributed reagents, materials, analysis tools. MP wrote the paper. EW, LS, AB, AK and KG revised the paper.

### Conflict of interest statement

The authors declare that the research was conducted in the absence of any commercial or financial relationships that could be construed as a potential conflict of interest.

## Supplementary material

Supplementary Movies for high-resolution, high-frame-rate videos of individual wild-type **(Video 1)**, *fcpB*^−^ mutant **(Video 2)**, and *fcpB*^−/+^ complemented leptospires **(Video 3)**, moving translationally in viscous liquid media (1% methyl-cellulose).

## References

[B1] Charon N. W. Goldstein S. F. (2002). Genetics of motility and chemotaxis of a fascinating group of bacteria: the spirochetes. Annu. Rev. Genet. 36, 47–73. 10.1146/annurev.genet.36.041602.134359 12429686

[B2] Costa F. Hagan J. E. Calcagno J. Kane M. Torgerson P. Martinez-Silveira M. S.. (2015). Global Morbidity and Mortality of Leptospirosis: a systematic Review. PLoS Negl. Trop. Dis. 9:e0003898. 10.1371/journal.pntd.0003898 26379143 PMC4574773

[B3] Fontana C. Lambert A. Benaroudj N. Gasparini D. Gorgette O. Cachet N.. (2016). Analysis of a spontaneous non-motile and avirulent mutant shows that FliM is Required for full endoflagella assembly in *Leptospira interrogans*. PLoS ONE 11:e0152916. 10.1371/journal.pone.0152916 27044038 PMC4820103

[B4] Fouts D. E. Matthias M. A. Adhikarla H. Adler B. Berg D. E. Bulach D.. (2016). What makes a bacterial species pathogenic? Comparative genomic analysis of the genus *Leptospira* PLoS Negl. Trop. Dis. 10:e0004403. 10.1371/journal.pntd.0004403 26890609 PMC4758666

[B5] Goldstein S. F. Charon N. W. (1988). Motility of the spirochete *Leptospira*. Cell Motil. Cytoskeleton 9, 101–110. 10.1002/cm.970090202 3282685

[B6] Goldstein S. F. Charon N. W. (1990). Multiple-exposure photographic analysis of a motile spirochete. Proc. Natl. Acad. Sci. U.S.A. 87, 4895–4899. 10.1073/pnas.87.13.4895 2367518 PMC54227

[B7] Lambert A. Picardeau M. Haake D. A. Sermswan R. W. Srikram A. Adler B. (2012). FlaA proteins in *Leptospira interrogans* are essential for motility and virulence but are not required for formation of the flagellum sheath. Infect. Immun. 80, 2019–2025. 10.1128/IAI.00131-12 22451522 PMC3370569

[B8] Li C. Sal M. Marko M. Charon N. W. (2010). Differential regulation of the multiple flagellins in spirochetes. J. Bacteriol. 192, 2596–2603. 10.1128/JB.01502-09 20304988 PMC2863563

[B9] Liao S. Sun A. Ojcius D. M. Wu S. Zhao J. Yan J. (2009). Inactivation of the *fliY* gene encoding a flagellar motor switch protein attenuates mobility and virulence of *Leptospira interrogans* strain Lai. BMC Microbiol. 9:253. 10.1186/1471-2180-9-253 20003186 PMC3224694

[B10] Macnab R. M. (2003). How bacteria assemble flagella. Annu. Rev. Microbiol. 57, 77–100. 10.1146/annurev.micro.57.030502.090832 12730325

[B11] Malmström J. Beck M. Schmidt A. Lange V. Deutsch E. W. Aebersold R. (2009). Proteome-wide cellular protein concentrations of the human pathogen *Leptospira interrogans*. Nature 460, 762–765. 10.1038/nature08184 19606093 PMC2723184

[B12] Mastronarde D. N. (2005). Automated electron microscope tomography using robust prediction of specimen movements. J. Struct. Biol. 152, 36–51. 10.1016/j.jsb.2005.07.007 16182563

[B13] Pallen M. J. Penn C. W. Chaudhuri R. R. (2005). Bacterial flagellar diversity in the post-genomic era. Trends Microbiol. 13, 143–149. 10.1016/j.tim.2005.02.008 15817382

[B14] Picardeau M. (2008). Conjugative transfer between *Escherichia coli* and *Leptospira* spp. as a new genetic tool. Appl. Environ. Microbiol. 74, 319–322. 10.1128/AEM.02172-07 17993560 PMC2223205

[B15] Picardeau M. (2017a). Toolbox of molecular techniques for Studying *Leptospira* spp. Curr. Top. Microbiol. Immunol. [Epub ahead of print]. 10.1007/82_2017_45 28849314

[B16] Picardeau M. (2017b). Virulence of the zoonotic agent of leptospirosis: still *terra incognita*? Nat. Rev. Microbiol. 15, 297–307. 10.1038/nrmicro.2017.5 28260786

[B17] Picardeau M. Brenot A. Saint Girons I. (2001). First evidence for gene replacement in *Leptospira* spp. Inactivation of *L. biflexa flaB results* in non-motile mutants deficient in endoflagella. Mol. Microbiol. 40, 189–199. 10.1046/j.1365-2958.2001.02374.x 11298286

[B18] Qin Z. Lin W. T. Zhu S. Franco A. T. Liu J. (2016). Imaging the motility and chemotaxis machineries in *Helicobacter pylori* by cryo-electron tomography. J. Bacteriol. 199:e00695-16. 10.1128/JB.00695-16 PMC5237115 27849173

[B19] San Martin F. Mechaly A. E. Larrieux N. Wunder E. A. Jr. Ko A. Picardeau M.. (2017). Crystallization of FcpA from Leptospira, a novel flagellar protein that is essential for pathogenesis. Acta Crystallogr. F Struct. Biol. Commun. 73(Pt 3), 123–129. 10.1107/S2053230X17002096 28291747 PMC5349305

[B20] Scheres S. H. (2012). A Bayesian view on cryo-EM structure determination. J. Mol. Biol. 415, 406–418. 10.1016/j.jmb.2011.11.010 22100448 PMC3314964

[B21] Slamti L. Picardeau M. (2012). Construction of a library of random mutants in the spirochete *Leptospira biflexa* using a mariner transposon, in Mobile Genetic Elements, ed Bigot Y. (Totowa, NJ: Humana Press), 169–176. 10.1007/978-1-61779-603-6_9 22367871

[B22] Stewart P. E. Carroll J. A. Olano L. R. Sturdevant D. E. Rosa P. A. (2015). multiple posttranslational modifications of *Leptospira biflexa* proteins as revealed by proteomic analysis. Appl. Environ. Microbiol. 82, 1183–1195. 10.1128/AEM.03056-1 26655756 PMC4751834

[B23] Takabe K. Kawamoto A. Tahara H. Kudo S. Nakamura S. (2017). Implications of coordinated cell-body rotations for *Leptospira* motility. Biochem. Biophys. Res. Commun. 491, 1040–1046. 10.1016/j.bbrc.2017.08.007 28780349

[B24] Tang G. Peng L. Baldwin P. R. Mann D. S. Jiang W. Rees I.. (2007). EMAN2: an extensible image processing suite for electron microscopy. J. Struct. Biol. 157, 38–46. 10.1016/j.jsb.2006.05.009 16859925

[B25] Torgerson P. R. Hagan J. E. Costa F. Calcagno J. Kane M. Martinez-Silveira M. S.. (2015). Global burden of Leptospirosis: estimated in terms of disability adjusted life years. PLoS Negl. Trop. Dis. 9:e0004122. 10.1371/journal.pntd.0004122 26431366 PMC4591975

[B26] Wolgemuth C. W. (2015). Flagellar motility of the pathogenic spirochetes. Semin. Cell Dev. Biol. 46, 104–112. 10.1016/j.semcdb.2015.10.015 26481969 PMC4994469

[B27] Wunder E. A. Jr. Figueira C. P. Santos G. R. Lourdault K. Matthias M. A. Vinetz J. M. (2016a). Real-Time PCR reveals rapid dissemination of *Leptospira interrogans* after intraperitoneal and conjunctival inoculation of hamsters. Infect. Immun. 84, 2105–2115. 10.1128/IAI.00094-16 27141082 PMC4936353

[B28] Wunder E. A. Jr. Figueira C. P. Benaroudj N. Hu B. Tong B. A. Trajtenberg F.. (2016b). A novel flagellar sheath protein, FcpA, determines filament coiling, translational motility and virulence for the *Leptospira* spirochete. Mol. Microbiol. 101, 457–470. 10.1111/mmi.13403 27113476 PMC4979076

[B29] Yonekura K. Maki-Yonekura S. Namba K. (2003). Complete atomic model of the bacterial flagellar filament by electron cryomicroscopy. Nature 424, 643–650. 10.1038/nature01830 12904785

[B30] Zhang K. (2016). Gctf: real-time CTF determination and correction. J. Struct. Biol. 193, 1–12. 10.1016/j.jsb.2015.11.003 26592709 PMC4711343

[B31] Zhao X. Norris S. J. Liu J. (2014). Molecular architecture of the bacterial flagellar motor in cells. Biochemistry 53, 4323–4333. 10.1021/bi500059y 24697492 PMC4221660

[B32] Zhu S. Nishikino T. Hu B. Kojima S. Homma M. Liu J. (2017). Molecular architecture of the sheathed polar flagellum in *Vibrio alginolyticus*. Proc. Natl. Acad. Sci. U.S.A. 114, 10966–10971. 10.1073/pnas.1712489114 28973904 PMC5642721

